# Effects of heat stress and rumen-protected fat supplementation on growth performance, rumen characteristics, and blood parameters in growing Korean cattle steers

**DOI:** 10.5713/ajas.18.0725

**Published:** 2019-01-02

**Authors:** Hyeok Joong Kang, Min Yu Piao, Seung Ju Park, Sang Weon Na, Hyun Jin Kim, Myunggi Baik

**Affiliations:** 1 Department of Agricultural Biotechnology and Research Institute of Agriculture and Life Sciences, College of Agriculture and Life Sciences, Seoul National University, Seoul 08826, Korea; 2 Feed Research Institute, Chinese Academy of Agricultural Sciences, Beijing 100081, China; 3 Institute of Green Bio Science and Technology, Seoul National University, Pyeongchang 25354, Korea

**Keywords:** Ambient Temperature, Beef Cattle, Blood Metabolites, Growth, Heat Stress, Rumen-protected Fat

## Abstract

**Objective:**

This study was performed to evaluate whether hot temperature and rumen-protected fat (RPF) supplementation affect growth performance, rumen characteristics, and serum metabolites in growing stage of Korean cattle steers.

**Methods:**

Twenty Korean cattle steers (230.4±4.09 kg of body weight [BW], 10.7±0.09 months of age) were divided into a conventional control diet group (n = 10) and a 0.8% RPF supplementation group (n = 10). Steers were fed 1.5% BW of a concentrate diet and 4 kg of tall fescue hay for 16 weeks (July 10 to August 6 [P1], August 7 to September 3 [P2], September 4 to October 1 [P3], October 2 to 30 [P4], of 2015).

**Results:**

The mean temperature-humidity index (THI) was higher (p<0.001) in P1 (76.8), P2 (76.3), and P3 (75.9) than in P4 (50.9). The mean THI of P1–3 were within the alert heat stress (HS) category range according to previously reported categories for feedlot cattle, and the mean THI of P4 was under the thermo-neutral range. Neither month nor RPF supplementation affected (p>0.05) average daily gain and gain to feed ratio. Month and RPF supplementation affected concentrations of glucose, albumin, and high-density lipoprotein (HDL); those of albumin and glucose tended to decrease (p<0.10), but HDL concentration increased (p<0.01) by RPF supplementation. Neither month nor RPF affected (p>0.05) ruminal pH, NH_3_-N, and volatile fatty acid concentrations, whereas the C2:C3 ratio was affected (p<0.05) by month.

**Conclusion:**

Korean cattle may not have been significantly affected by alert HS during the growing stage. Growth performance was higher during hotter months, although some changes in blood metabolites were observed. The RPF supplementation affected some blood lipids and carbohydrate metabolites but did not affect growth performance.

## INTRODUCTION

Animal productivity (e.g., growth performance, milk yield, or meat quality) is affected by various factors, including genetic, management, nutritional, and environmental factors [1]. Heat stress (HS) is an important stress type that can negatively affect dry matter intake (DMI), growth performance, gain to feed ratio (G:F ratio), overall health, milk production, or reproduction [2,3]. Measuring body temperature or respiration rate is the most appropriate method to determine whether cattle have been exposed to HS in a hot environment, but it is less efficient in commercial farms with large numbers of cattle [4,5]. Therefore, the temperature-humidity index (THI) was developed as an indicator to determine HS [6,7]. The HS can affect animal energy metabolism, which reduces metabolic heat production to maintain normal body temperature [8]. These changes inevitably affect energy metabolites in the blood. Previous studies have reported changes in beef and dairy cattle blood parameters and reduced productivity under hot climatic conditions [9,10]. In turn, changes in metabolic status among cattle under HS are associated with hormonal imbalance [11]. These sequential metabolic responses cause cattle to waste energy in maintaining homeostasis that could contribute to weight gain or other production performance. HS can also depress rumination, reticulo-rumen motility, and ruminal activity, which slow the fractional passage rate of digesta through the gastrointestinal tract [12]. Depressed rumen activity due to HS largely reflects decreases in DMI and volatile fatty acid (VFA) production in the rumen [12,13]. To alleviate negative HS responses, providing additional energy should be considered.

Several studies have attempted to determine optimal nutrition to decrease HS and cold stress and increase animal productivity, including dietary fat supplementation [14–16]. Ruminal activity is depressed by heat; therefore, the addition of unprotected forms of fat may decrease rumen fermentation and adversely affect rumen motility. Thus, in this study, we used dietary rumen-protected fat (RPF) as a supplementary source of fat in a concentrate. This diet was designed to increase energy absorption in the small intestine while minimizing fat fermentation in the rumen, effectively compensating for increased maintenance energy under HS conditions. Although dietary RPF has been widely used in previous studies [16,17], the effects of RPF supplementation on growth performance, rumen characteristics, and blood parameters in growing stage of Korean cattle under HS conditions remain largely unknown.

## MATERIALS AND METHODS

### Animals and feeding trials

All experimental procedures involving animals were approved by the Seoul National University Institutional Animal Care and Use Committee (SNUIACUC), Republic of Korea, and conducted in accordance with the Animal Experimental Guidelines of the SNUIACUC. This study was conducted at the University Animal Farm of the College of Agriculture and Life Sciences, Pyeongchang Campus of Seoul National University, South Korea.

Twenty Korean cattle steers were used in a feedlot trial; these steers had an average age of 10.7±0.09 months and weight of 230.4±4.09 kg. The steers were fed commercial growing stage concentrate using an automatic feeding station (DeLaval Alpro system; DeLaval, Tumba, Sweden) and timothy hay, following a conventional feeding program. Drinking water was provided freely. During a 2-week adaptation period before the experiment, all animals were fed an experimental control concentrate (approximately 1.5% body weight [BW] per animal) and timothy hay (5 kg/d/head). Steers were assigned to one of two treatments: control and RPF supplementation. The RPF is prilled form of palm oil, as described [18], and purchased from Ecolex SDN. BHD (Pulau Indah, Selangor, Malyasia). The RPF was composed of 99.63% free fatty acids, including 85.48% palmitic acid (C 16:0), 7.05% oleic acid (C 18:1), 3.45% myristic acid (C 14:0), 1.64% linoleic acid (C 18:2), and 1.04% lauric acid (C 12:0), with an energy density of 9,316 kcal/kg (Haneol Corp., Anseong, Korea). Table 1 lists the formula and chemical compositions of the experimental diets. Steers were fed a concentrate (1.5% BW) using an automatic feeding station and a timothy hay diet (5 kg/d) for 16 weeks (July 10 to August 6 [P1], August 7 to September 3 [P2], September 4 to October 1 [P3], and October 2 to 30 [P4], of 2015). The daily concentrate intake was automatically recorded online using a computer with the DeLaval Alpro system (Sweden). Equal amounts of roughage were provided twice daily (08:00 and 18:00) and residual roughage was weighed before the morning feeding. Thus, the ratio of concentrate to hay was not fixed, and the ratio was close to 1.0 in fed base. Concentrate and timothy hay samples were collected weekly and stored at −20°C until analysis. The BW was measured before the morning feeding on the start day and at 4-week intervals thereafter.

### Chemical composition analysis

The chemical compositions (dry matter, crude protein, ether extract, ash, calcium, and phosphorus) of the concentrate and timothy were determined using the Association of Official Analytical Chemists (AOAC) method [19]. The neutral and acid detergent fiber contents of the experimental concentrate and timothy were analyzed using the sequential method with an ANKOM200 Fiber Analyzer (Ankom Technology Corp., Macedon, NY, USA) and reagents described by Van Soest et al [20].

### Blood collection and ambient temperature measurements

Blood was collected before feeding (after 9 h of fasting) at approximately 09:00 on the start date and at 4-week intervals thereafter. Blood was collected via jugular venipuncture with a non-heparinized vacutainer (20 mL; Becton-Dickinson, Franklin Lakes, NJ, USA). Serum was separated by centrifugation at 1,500×*g* at 4°C for 15 min and stored at −80°C until analysis.

Ambient (inside of the barn) and climate (outside of the barn) temperatures, and relative humidity inside and outside the barn, were recorded at 1-h intervals using four HOBO data loggers (Onset Computer Corp., Bourne, MA, USA). Monthly average values of minimum, mean, and maximum temperatures and humidity values were calculated using daily data. The THI (modified from [21]) was calculated as previously described [7] from automatically recorded ambient or climate temperature and humidity data. The equation is as follows.

THI=0.8×temperature+[(relative humidity×0.01)×(temperature-14.4)]+46.4

The experimental farm was covered by a roof and both side doors were closed; thus, the animals were raised indoors. Steers could access the feeding station and roughage bucket freely, but they were not allowed to move outside the barn. As such, we ignored any effects of rain, direct sunlight, or wind. Thus, humidity and temperature, subsumed within the THI, were the major climate factors in this study.

### Rumen fluid collection and analysis

Following blood collection, rumen fluid was collected after 3 h of feeding using the oral stomach tube method, as described by Shen et al [22]. Rumen fluid pH was measured immediately using a pH meter (Ohaus Corp., Parsippany, NJ, USA). The 1 mL VFA rumen fluid samples were mixed with 0.2 mL of 25% meta-phosphoric acid and storage at −20°C until analysis. An additional 30 mL of rumen fluid was stored at −20°C for ruminal ammonia nitrogen (NH_3_-N) analysis. NH_3_-N concentrations were determined using a modified colorimetric method [23]. The VFA concentrations were determined using an Agilent Tech 7890A gas chromatograph (Hewlett Packard, Waldbronn, Germany) with a Supelco fused silica capillary column (30 m×0.25 mm×0.25 μm).

### Blood analysis

Analytical reagents for albumin, glucose, triglyceride (TG), high-density lipoprotein (HDL), low-density lipoprotein, cholesterol, glutamic oxaloacetic transaminase (GOT), and glutamic pyruvate transaminase (GPT) were purchased from JW Medical (Seoul, Korea). Analytical reagents for non-esterified fatty acid (NEFA) analysis were purchased from Wako Pure Chemical (Osaka, Japan). All parameters were analyzed using an automated chemistry analyzer (Hitachi 7180; Hitachi, Tokyo, Japan). All analyses were validated in our laboratory, as previously reported [9].

### Statistical analysis

Differences in climate parameters among months were analyzed using one-way analysis of variance (ANOVA). Differences in growth performance, blood parameters, and rumen fluid parameters due to month and dietary treatment were analyzed using repeated-measures two-way ANOVA. The statistical model included month, diet, and their interaction. Significance was determined at a level of p<0.05. All statistical tests were performed using the R Studio for Windows software (R Studio, Boston, MA, USA).

## RESULTS AND DISCUSSION

### Climate conditions

The mean (P1, 76.8; P2, 76.3; P3, 75.9) and maximum (P1, 81.8; P2, 81.3; P3, 80.3) indoor THI in July (P1), August (P2), and September (P3) were higher (p<0.001) than those in October (P4: mean, 50.9; maximum, 63.7; Table 2). The temperatures outside of the experimental barn are presented in Table 2. In a previous study, HS in beef cattle was categorized as thermo-neutral (<74), alert (75 to 78), danger (79 to 83), and emergency (>84) at low wind speed and solar radiation conditions [7]. Therefore, the ambient mean THI values for P1, P2, and P3 in this study were considered to represent alert HS conditions; however, P4 was considered as thermo-neutral condition. We also measured the average temperature and relative humidity at blood and rumen fluid collection time (0800 to 1200), then calculated THI of each sampling day. The THIs were 78.3, 76.3, 68.6, 55.3, and 42.2 on July 10, August 7, September 3, October 1, and October 30, respectively. The first two blood sampling days were classified as alert HS condition, while the other 3 days were classified as thermo-neutral conditions.

### Growth performance

The BW was higher (p<0.001) in P4 than in P1, reflecting animal age. The daily concentrate intake was higher (p = 0.03) in P4 than in P1 (Table 3). In this study, the daily allowance of concentrate was set at 1.5% BW and adjusted each month based on BW. Thus, higher concentrate intake during P4 reflects the increased concentrate allowance corresponding to higher BW during the experimental periods. Daily forage intake was lower (p<0.05) in P1 than in all other months. The percentage intake of concentrate, forage, and total feed relative to the offered amount were lower (p<0.05) in the hotter months, although RPF did not affect (p>0.05) the percentage intake (Table 3). Under hot condition, DMI generally decreases [2]. RPF supplementation tended (p = 0.07) to decrease concentrate intake and decreased (p<0.01) forage intake. In a dairy study, DMI decreased following supplementation with protected lipids [24]. Average daily gain and G:F ratio were higher (p<0.001) during the hotter months. The RPF supplementation did not affect (p>0.05) average daily gain or the G:F ratio (Table 3). Considering the decrease in percentage of DMI and increase in growth performance during hotter months observed in this study, factors other than THI may have affected growth performance, such as heredity, diet, or age [25]. In this study, genetic factors and diet were similar among experimental months; however, age differed between periods. Goonewardene et al [25] showed that the highest growth rate occurred in cattle aged about 0 to 300 days; subsequently, growth rates decreased as age increased. Thus, the higher weight gain and better G:F ratio during hotter months observed in this study may have been due to age instead of THI.

### Rumen volatile fatty acids and NH_3_-N

Ruminal pH was not changed (p>0.05) by temperature or RPF supplementation. Ruminal NH_3_-N and all VFA parameters (C2, C3, C4, iso-C4, C5, iso-C5, and total VFAs) were unchanged (p>0.05) by temperature or RPF supplementation (Table 4). The C2:C3 ratio was lower (p<0.05) on the experimental starting day than in all other periods, with the highest THI (Table 4). Similarly, HS resulted in depression of ruminal VFAs and C2:C3 ratio [26,27]. The depression of ruminal VFAs under hot conditions resulted from greater VFA metabolism and utilization from arterial blood [28].

### Blood metabolites

Circulating glucose was lower (p<0.001) under hotter conditions and was decreased (p = 0.08) by RPF supplementation (Table 5). Previous studies have also reported lower blood glucose concentrations under higher temperatures [9,29]. Decreased blood glucose under hotter conditions can be explained by altered homeostasis of hormones such as insulin, or rapid utilization of blood glucose due to increased respiratory rate as a result of hyperthermia [29,30]. Indeed, glucose can become a primary energy source for heat-stressed cattle [31]. Blood NEFA concentrations were higher (p<0.001) only on the starting day, and NEFAs were not changed (p>0.05) by RPF supplementation (Table 5). Blood NEFA concentrations in dairy cattle often increase when feed intake cannot support their energy requirements, requiring the mobilization of NEFAs by lipolysis of fat depots to support energy demand [32]. In this study, the highest NEFA concentration was observed at the highest THI on the experimental starting day, presumably due to status changes among hormones [11], such as catecholamine and glucocorticoids, which typically promote adipocyte lipolysis and NEFA mobilization [29].

Blood HDL, TG, and cholesterol concentrations were lower (p<0.05) during hotter periods than in thermo-neutral periods (Table 5). Similarly, our previous study showed that cholesterol and HDL concentrations were lower (p<0.05) under higher temperature conditions [9]. Ronchi et al [33] suggested that lower blood cholesterol results from increased lipid utilization by peripheral tissues. Moreover, HS increases adipose tissue lipoprotein lipase [34], indicating that adipose tissue of animals under HS has increased capacity to liberate fatty acids from circulating TGs for storage [35]. Decreased HDL concentrations in beef cattle under HS has not been demonstrated previously, but increased lipoprotein lipase could be an explanation for this observation. HDL concentrations were increased (p<0.01) by RPF supplementation (Table 5), but TG and cholesterol were not affected (p>0.05). Lee et al [36] reported that rumen-protected oleic acid in the diet increases blood HDL concentrations, which may be derived from absorbed RPF in the small intestine.

Blood GPT concentrations were lower (p<0.05) during hotter periods than in the thermo-neutral period; however, they were not affected (p>0.05) by RPF supplementation (Table 5). As in our study, previous studies showed that heat-exposed ewes had lower GOT and GPT levels, probably due to a reduction in thyroid hormone secretion, which decreased endogenous body heat production [37,38]. Little information is available on the effects of dietary fat supplementation on blood GOT and GPT in beef cattle. Blood albumin concentration showed variation (p<0.001) among months and tended to decrease (p = 0.08) by RPF supplementation (Table 5). The lowest blood albumin concentration was recorded during the hottest month (August 7); this result was similar to that of our previous Korean cattle study and was perhaps caused by physiological activity maintaining blood osmolality under hotter conditions [9]. The effect of RPF supplementation on serum albumin concentration in beef cattle remains unclear.

## CONCLUSION

Growth performance (average daily gain and G:F ratio) did not worsen during hotter months. Rumen fermentation parameters such as ruminal pH, NH_3_-N, and VFA concentrations were not significantly affected by HS conditions or RPF supplementation, although the C2:C3 ratio differed between months. Blood parameters were more sensitive to HS conditions than to rumen parameters. Serum TG, cholesterol, HDL, and glucose were lower under hotter conditions, and other serum parameters such as NEFA, GOT, and GPT were affected by month. Serum glucose, HDL, and albumin were affected by RPF supplementation. Thus, Korean cattle in growing stage may not be significantly affected by HS conditions, although rumen fermentation and blood parameters were partially affected in the current study. RPF supplementation did not affect growth performance or major rumen VFA concentrations; however, RPF supplementation affected glucose and HDL concentrations. Further research is warranted to determine which HS conditions significantly affect the growth performance of Korean cattle during the growing stage.

## Figures and Tables

**Table 1:** Ingredient of concentrate and composition of experimental diets for Korean cattle steers during July through October of 2015

Items	Control concentrate	RPF concentrate
Ingredient of concentrate		
Ground corn	17.02	16.78
Wheat bran	20.00	20.00
Rice bran	5.00	5.00
Salt	0.37	0.37
Alfalfa–pellet	1.50	1.50
Molasses	3.80	3.80
Ammonium chloride	0.50	0.50
Palm meal	10.00	10.00
Condensed molasses soluble	1.50	1.50
Coconut meal	10.00	10.00
Distiller’s dried grains with solubles	4.71	9.34
Live yeast	0.01	0.01
Corn flour	4.20	4.00
Soy hull	16.84	12.34
Rumen-protected fat	0.00	0.80
Limestone	3.04	3.06
Antimicrobial agent1)	0.05	0.05
Barley stone	1.20	0.69
Mineral/vitamin premix2)	0.26	0.26
Total	100.00	100.00
Chemical composition (% DM)		
Concentrate		
DM	89.44	89.25
CP	15.00	15.00
EE	4.10	5.23
Ash	9.47	8.83
NDF	24.43	24.23
ADF	35.91	35.11
NFC	30.98	30.75
Ca	1.05	0.95
P	0.40	0.48
TDN3) (%)	70.39	72.50
ME4) (Mcal/kg)	2.69	2.79
NE5) (Mcal/kg)	1.15	1.22
Timothy hay (% DM)		
DM	90.63	
CP	6.06	
EE	1.55	
Ash	5.89	
Ca	0.35	
P	0.19	
ADF	66.76	
NDF	38.50	

RPF, rumen-protected fat; DM, dry matter; CP, crude protein; EE, ether extract; NDF, neutral detergent fiber; ADF, acid detergent fiber; NFC, non-fiber carbohydrate; TDN, total digestible nutrients; ME, metabolizable energy; NE, net energy.

1) The experiment was conducted during summer season, thus “antimicrobial agent” was used to prevent the concentrate from being decayed or fungal infection. Antimicrobial agent contained 65% of propionic acid and 35% of sorbic, acetic, benzoic, and phosphoric acids (Kemin Industry, Des Moines, IA, USA).

2) Mineral and vitamin premix contained vit. A, 2,650,000 IU; vit. D_3_, 530,000 IU; vit. E, 1,050 IU; niacin, 10,000 mg; Mn, 4,400 mg; Zn, 4,400 mg; Fe, 13,200 mg; Cu, 2,200 mg; iodine, 440 mg; Co, 440 mg/kg of Grobic–DC. Grobic–DC was provided by Bayer Health Care (Leverkusen, Germany).

3) TDN (%) = NFC+CP+[(EE–1)×2.25]+NDF–7 [39].

4) ME = [1.01×(DE)–0.45]+0.0046×(EE–3) [39].

5) NE = 1.42 ME–0.174 ME^2^+0.0122 ME^3^–1.65 [39].

**Table 2:** Mean, maximum, and minimum values of ambient temperature, climate temperature, relative humidity, and temperature-humidity index1) at July to October of 2015

Items	July (P1)2)	August (P2)3)	September (P3)4)	October (P4)5)	SE	p-value
Ambient temperature (°C)						
Mean	24.9^a^	23.4^a^	18.4^b^	9.8^c^	0.55	<0.001
Maximum	30.8^a^	32.9^a^	28.8^ab^	19.8^c^	0.65	<0.001
Minimum	21.3^a^	18.1^b^	11.7^c^	3.75^d^	0.66	<0.001
Climate temperature (°C)						
Mean	24.2^a^	21.4^b^	16.7^c^	10.4^d^	0.57	<0.001
Maximum	30.2^a^	28.1^a^	24.9^b^	17.5^c^	0.63	<0.001
Minimum	20.6^a^	17.0^b^	11.0^c^	5.3^d^	0.64	<0.001
Ambient relative humidity (%)						
Mean	100.4^a^	67.0^b^	71.3^b^	72.7^b^	2.41	<0.001
Maximum	120.3^a^	97.9^b^	102.3^b^	99.9^b^	1.68	<0.001
Minimum	71.4^a^	68.0^a^	64.8^ab^	30.4^b^	3.90	<0.001
Climate relative humidity (%)						
Mean	102.1^a^	102.8^a^	97.4^b^	83.0^c^	1.33	<0.001
Maximum	122.9^a^	127.6^a^	127.6^a^	106.6^b^	1.14	0.003
Minimum	70.9^a^	71.1^a^	72.3^a^	49.8^b^	1.95	<0.001
Ambient THI						
Mean	76.8^a^	76.3^a^	75.9^a^	50.9^b^	0.94	<0.001
Maximum	81.8^a^	81.3^a^	80.9^a^	63.7^b^	0.83	<0.001
Minimum	72.0^a^	71.5^a^	71.1^a^	38.9^b^	1.25	<0.001
Climate THI						
Mean	70.9^a^	71.1^a^	72.3^a^	51.4^b^	1.01	<0.001
Maximum	81.1^a^	80.7^a^	80.3^a^	61.6^b^	0.86	<0.001
Minimum	70.6^a^	70.1^a^	69.8^a^	40.9^b^	1.28	<0.001

THI, temperature-humidity index; SE, standard error.

1) THI = 0.8×temperature+[(relative humidity×0.01)×(temperature–14.4)]+46.4 [7].

2) July 10 to August 6 (4 weeks).

3) August 7 to September 3 (4 weeks).

4) September 4 to October 1 (4 weeks).

5) October 2 to October 30 (4 weeks).

a–d Mean values with different letters within same row differ (p<0.05).

**Table 3:** Growth performance of growing Korean cattle steers fed either control or RPF-supplemented diet during July through October of 2015

Items	July1)		August2)		September3)		October4)		SE	p-value		
				
	Control	RPF	Control	RPF	Control	RPF	Control	RPF		Month	Diet	Interaction
Age (mo)	10.6	10.7	11.5	11.6	12.5	12.6	13.4	13.5	0.35	<0.001	0.87	
Initial body weight (kg)	231.3	229.4	259.1	257.4	288.6	282.7	300.2	294.2	2.59	<0.001	0.45	0.68
Body weight (kg)	259.1	257.4	288.6	282.7	300.2	294.2	319.7	312.5	2.67	<0.001	0.51	0.34
Daily feed intake (kg, DM base)												
Total feed intake (kg/d)	6.93	6.84	7.48	7.36	8.00	7.80	8.32	8.03	0.29	<0.001	0.46	0.76
Percentage of total feed intake5) (%)	77.0	77.0	75.6	74.9	87.1	85.6	87.5	85.2	1.24	<0.001	0.62	0.72
Concentrate intake (kg/d)	3.18	3.13	3.58	3.59	3.93	3.79	4.22	4.05	0.24	<0.001	0.07	0.34
Percentage of concentrate intake6) (%)	85.4	84.7	75.4	75.7	93.8	92.7	93.8	91.9	1.76	0.009	0.81	0.86
Forage intake (g/d)	3.76	3.72	3.90	3.77	4.08	4.01	4.09	3.98	0.08	0.011	0.0014	0.09
Percentage of forage intake7) (%)	71.5	71.8	75.7	74.0	81.6	80.2	81.9	79.6	1.46	0.011	0.66	0.77
Average daily gain (kg/d)	0.99	1.00	1.05	0.90	0.75	0.76	0.70	0.65	0.01	<0.001	0.93	0.02
G:F ratio	0.144	0.147	0.142	0.123	0.094	0.099	0.085	0.083	0.0003	<0.001	0.42	0.03

N = 10/group.

RPF, rumen-protected fat; SE, standard error; DM, dry matter; G:F, gain to feed ratio.

1) July 10 to August 6 (4 weeks).

2) August 7 to September 3 (4 weeks).

3) September 4 to October 1 (4 weeks).

4) October 2 to October 30 (4 weeks).

5),6),7) Percentage of feed intake relative to the offered amount.

5) Total feed includes both concentrate and forage intake.

**Table 4:** Ruminal pH, VFAs and NH_3_-N of Korean cattle steers fed either control or RPF-supplemented diet during July through October of 2015 (after 3 h of feeding)

Items	July 10		August 7		September 4		October 2		October 30		SE	p-value		
					
	Control	RPF	Control	RPF	Control	RPF	Control	RPF	Control	RPF		Month	Diet	Interaction
THI1)	78.3		76.3		68.6		55.3		42.2					
pH	6.88	7.02	6.74	7.10	6.84	6.54	6.73	6.86	6.64	6.50	0.05	0.65	0.78	0.70
NH_3_-N (mg/dL)	11.7	12.7	8.60	8.66	11.8	10.2	10.9	9.91	8.48	9.08	0.51	0.50	0.59	0.77
C2 (mM)	67.3	77.2	66.3	65.4	58.6	59.9	69.8	60.5	69.7	65.3	1.44	0.32	0.39	0.82
C3 (mM)	25.7	27.2	19.3	15.4	17.2	22.2	19.2	18.7	26.9	20.4	0.69	0.54	0.62	0.73
Iso C4 (mM)	0.52	0.64	0.53	0.51	0.66	0.87	0.67	0.72	0.67	0.70	0.03	0.90	0.60	0.72
C2:C3 ratio	2.62	2.84	3.44	4.25	3.41	2.70	3.64	3.24	2.59	3.20	0.08	0.04	0.54	0.48
C4 (mM)	15.5	11.9	11.9	10.8	12.1	11.9	13.1	12.0	10.5	11.4	0.40	0.46	0.55	0.64
Iso C5 (mM)	0.80	0.89	1.21	0.82	0.99	0.84	0.76	0.65	0.84	1.09	0.04	0.44	0.29	0.37
C5 (mM)	1.54	1.22	1.07	1.60	1.14	0.95	1.17	1.44	1.06	1.17	0.02	0.81	0.60	0.45
Total VFA (mM)	111.4	119.1	100.3	94.5	90.7	96.7	104.7	94.0	109.7	100.1	1.79	0.57	0.84	0.64

N = 10/group.

VFA, volatile fatty acid; RPF, rumen-protected fat; SE, standard error; THI, temperature-humidity index.

1) THI = 0.8×temperature+[(relative humidity×0.01)×(temperature–14.4)]+46.4 [7]; THI calculated based on average temperature and humidity of sampling times of each day.

**Table 5:** Serum parameters of Korean cattle steers fed either control or RPF–supplemented diet during July through October of 2015 (before feeding)

Items	July 10		August 7		September 3		October 1		October 30		SE	p-value		
					
	Control	RPF	Control	RPF	Control	RPF	Control	RPF	Control	RPF		Month	Diet	Interaction
Glucose (mg/dL)	65.4	63.3	69.7	68.6	72.5	66.5	81.1	77.1	82.3	79.0	0.95	<0.001	0.08	0.72
NEFA (mg/dL)	526.5	393.8	246.9	196.9	265.3	241.6	255.1	253.9	258.3	302.7	13.9	<0.001	0.17	0.03
Triglyceride (mg/dL)	14.4	13.5	16.3	16.3	15.8	15.8	20.2	16.1	17.2	16.8	0.40	0.017	0.27	0.70
Cholesterol (mg/dL)	99.6	111.8	110.6	100.0	125.3	136.1	137.3	142.2	140.4	152.9	0.68	<0.001	0.14	0.72
HDL (mg/dL)	65.8	77.7	68.6	69.0	78.1	88.5	83.4	92.0	89.7	94.7	0.98	<0.001	0.006	0.67
GOT (IU/dL)	75.1	72.1	74.6	62.3	81.2	69.5	78.2	75.1	77.4	67.9	1.25	0.005	0.56	0.77
GPT (IU/dL)	21.9	23.5	24.1	24.7	28.1	28.5	27.7	30.4	27.2	26.4	0.37	<0.001	0.31	0.72
Albumin (mg/dL)	3.30	3.23	3.03	2.76	3.35	3.30	3.56	3.47	3.55	3.37	0.02	<0.001	0.08	0.81

N = 10/group.

RPF, rumen-protected fat; SE, standard error; NEFA, non-esterified fatty acid; HDL, high density lipoprotein; GOT, glutamic oxalacetic transaminase; GPT, glutamic pyruvate transaminase.
